# Generation of Mast Cells from Mouse Fetus: Analysis of Differentiation and Functionality, and Transcriptome Profiling Using Next Generation Sequencer

**DOI:** 10.1371/journal.pone.0060837

**Published:** 2013-04-03

**Authors:** Nobuyuki Fukuishi, Yuusuke Igawa, Tomoyo Kunimi, Hirofumi Hamano, Masao Toyota, Hironobu Takahashi, Hiromichi Kenmoku, Yasuyuki Yagi, Nobuaki Matsui, Masaaki Akagi

**Affiliations:** 1 Department of Pharmacology, Faculty of Pharmaceutical Sciences, Tokushima Bunri University, Tokushima, Japan; 2 Institute of Pharmacognosy, Tokushima Bunri University, Tokushima, Japan; National Institutes of Health, United States of America

## Abstract

While gene knockout technology can reveal the roles of proteins in cellular functions, including in mast cells, fetal death due to gene manipulation frequently interrupts experimental analysis. We generated mast cells from mouse fetal liver (FLMC), and compared the fundamental functions of FLMC with those of bone marrow-derived mouse mast cells (BMMC). Under electron microscopy, numerous small and electron-dense granules were observed in FLMC. In FLMC, the expression levels of a subunit of the FcεRI receptor and degranulation by IgE cross-linking were comparable with BMMC. By flow cytometry we observed surface expression of c-Kit prior to that of FcεRI on FLMC, although on BMMC the expression of c-Kit came after FcεRI. The surface expression levels of Sca-1 and c-Kit, a marker of putative mast cell precursors, were slightly different between bone marrow cells and fetal liver cells, suggesting that differentiation stage or cell type are not necessarily equivalent between both lineages. Moreover, this indicates that phenotypically similar mast cells may not have undergone an identical process of differentiation. By comprehensive analysis using the next generation sequencer, the same frequency of gene expression was observed for 98.6% of all transcripts in both cell types. These results indicate that FLMC could represent a new and useful tool for exploring mast cell differentiation, and may help to elucidate the roles of individual proteins in the function of mast cells where gene manipulation can induce embryonic lethality in the mid to late stages of pregnancy.

## Introduction

Mast cells are known to be intimately involved in allergic responses through an aggregation of surface-expressed FcεRI followed by a release of inflammatory mediators including histamine, prostaglandins and cytokines [1], [2]. Mast cells also generate a wide variety of chemical mediators by exposure of bacterial components [3], [4], and share many features with primary effector cells that belong to the innate and acquired immune system [5], [6]. Therefore, understanding the mechanisms underlying the functions of mast cells is crucial, not only for the elucidation of the pathogenesis of allergy, but also clarification of the overall immune system [6], [7]. Conventional or conditional gene inactivation or deletion is widely utilized for the investigation of protein function [8], [9], and these techniques are remarkably helpful for the analysis of protein properties in a wide variety of cells [10], [11]. In mast cells, MacNeil *et al* indicated using a MAPK kinase 3 (MKK3)-knockout mouse that MKK3 is closely associated with the production of IL-4 in mast cells through the marked decrease of early growth response-1 binding to the IL-4 promoter region [12]. In addition, Hu *et al* demonstrated using a p38MAPK knockout mouse that p38MAPK, which lies downstream of MKK3 and has been reported to regulate the production of inflammatory cytokines in mast cells [13], is also crucial for the regulation of mast cell differentiation and migration [14]. Although gene modification can be a powerful strategy for the elucidation of protein function in various cells, including mast cells, gene knockout is known to induce embryonic lethal phenotypes [15]. For instance, P38MAPKα knockout mice are known to be embryonic lethal and die in mid-gestation with defects in placental and embryonic vasculature [16]. In such cases of fetal death by gene manipulation, the functional analysis of proteins in mast cells is virtually impossible because both “isolated” and “generated” mast cells are derived from adult tissues; specifically, they are isolated from lung [17], skin [18], [19], tonsil [19] and peritoneal fluid [20], and are generated from bone marrow [21], peripheral blood [22] and umbilical cord blood [23]. In the present study, we generated mast cells from mouse fetal liver cells (FLMC) and compared the properties with bone marrow-derived mast cells (BMMC). We reveal that FLMC have almost the same properties as BMMC, and thus expand the possibilities for characterization of proteins in mast cells in cases where gene manipulation causes an embryonic lethal phenotype.

## Materials and Methods

### Animals

C57BL/6 mice (Japan SLC, Shizuoka, Japan) were used for all experiments. Animal studies were approved by the Animal Care and Use Committee of the Faculty of Pharmaceutical Sciences at Tokushima Bunri University.

### Preparation of Pokeweed mitogen conditioned medium (CM)

Conditioned medium from Pokeweed mitogen-stimulated spleen cells was prepared as previously described [24]. Briefly, spleen cells from C57BL/6 mice were cultured at a density of 2×10^6^ cells/ml in RPMI 1640 medium (Gibco, Grand Island, NY) supplemented with 2 mM L-glutamine (Gibco), penicillin-streptomycin (Gibco), 10% fetal calf serum (Equitech Bio Inc., Kerrville TX), 8.8 µg/ml 2-mercaptoethanol (Kanto Chemical Co. Inc., Japan) and 3.3 µg/ml lectin from *Phytolacca americana* (Pokeweed mitogen; Sigma, St. Louis MO). After 48 hr, the cell suspension was centrifuged at 2,000 rpm for 10 min at 4°C, and the supernatant was removed and stored at –20°C. Immediately before use, the supernatant was thawed.

### Cell culture

Bone marrow cells were prepared from thighbones and shinbones of C57BL/6 mice as previously described [25]. Additionally, liver from C57BL/6 mouse embryos at embryonic age 12.5 days were dissected out, and then the liver was gently crushed through a fine mesh. During the first 21 days, both cell preparations were cultured in RPMI 1640 medium (Gibco) supplemented with 1 mM pyruvate (Gibco), penicillin-streptomycin (Gibco), non-essential amino acid (Gibco), 10% fetal calf serum (FBS; Equitech Bio Inc.), 100 µg/ml 2-mercaptoethanol (Kanto Chemical Co. Inc., Japan) and 10% CM. From 22 days on, the cells were continuously cultured in RPMI 1640 medium (Gibco) supplemented with 1 mM pyruvate (Gibco), penicillin-streptomycin (Gibco), non-essential amino acids (Gibco), 10% FBS (Equitech Bio Inc.), 100 µg/ml 2-mercaptoethanol (Kanto Chemical Co. Inc.) and 5 ng/ml of interleukin-3 (Miltenyi Biotec, Germany).

### Fractionation of lineage-negative cells

The lineage-negative (lin^-^) cells were isolated by depletion of cells expressing lineage antigens (CD5, B220, CD11b, Gr-1, 7–4, and Ter-119) using a commercial kit (Miltenyi Biotec). Briefly, freshly isolated bone marrow cells or fetal liver cells were incubated with a mixture of biotin-conjugated antibodies against the above-described lineage antigens for 10 min at 4°C. Next, the anti-biotin-conjugated microbeads were added to the mixture and incubated for 15 min at 4°C followed by washing twice with Dulbecco’s PBS containing 2 mM EDTA and 0.5% FBS (MACS buffer). The washed cells were then suspended in MACS buffer and applied to a magnetic column under a magnetic field. The effluent was collected as lin^-^ cells.

### Flow cytometry for detection of FcεRI and c-Kit

The surface expression levels of high affinity IgE receptor, FcεRI and c-Kit were monitored at 7-day intervals by flow cytometry as previously described [5]. Briefly, 1×10^5^ bone marrow-derived cells and/or fetal liver-derived cells were incubated with anti-CD16/CD32 mAb (2.4G2: BD Bioscience, San Jose, CA) for Fc receptor blocking followed by incubation with the mixture of phycoerythrin-conjugated anti mouse c-Kit antibody (2B8: BD Bioscience) and fluorescein isothiocyanate-conjugated anti mouse FcεRIα antibody (MAR-1: eBioscience, San Diego, CA). The stained cells were washed with RPMI 1640 containing 2% FBS twice, and analyzed by flow cytometry (Epics Altra; Beckman Coulter, Indianapolis IN).

### Flow cytometry for detection of Sca-1 and c-Kit expression in lin^-^ cells

The surface expression of Sca-1 and c-Kit were analyzed by flow cytometry. Briefly, 2×10^5^ lin^-^ bone marrow-derived cells and/or fetal liver-derived cells were incubated with an anti-CD16/CD32 mAb (2.4G2: BD Bioscience) for Fc receptor blocking followed by incubation with a mixture of phycoerythrin-conjugated anti-mouse c-Kit antibody (2B8: BD Bioscience) and fluorescein isothiocyanate-conjugated anti-mouse Sca-1 antibody (D7: Santa Cruz Biotechnology, Santa Cruz, CA). The stained cells were washed with RPMI 1640 containing 2% FBS twice, and analyzed by flow cytometry (Epics Altra; Beckman Coulter).

### Alcian blue and safranin O staining

After 5–6 weeks of culture, BMMC and FLMC were stained with alcian blue (Wako, Japan) and safranin O (Sigma) as previously described [26]. Briefly, cells were smeared onto 3-aminopropyltriethoxysilane-coated glass slides, and lightly heated and ethanol-fixed, and then stained for 5 min with 1.0% alcian blue at pH 2.5 in 1% acetic acid, followed by staining for 15 min with 0.1% safranin O.

### Transmission electron microscopy

At 35 days of culture, BMMC and FLMC were fixed and processed for transmission electron microscopy as previously described [27]. Thick sections were scanned after toluidine blue staining by light microscopy at x 1,000 to estimate the number of cells in these preparations. Thin sections of the same specimens were then examined using a transmission electron microscope (H7650; Hitachi, Tokyo, Japan). The intracellular organelles such as cytoplasmic granules were photographed and printed at x18,000.

### Degranulation, histamine release and histamine content measurement

For IgE sensitization, BMMC and FLMC, which were cultured for 5–6 weeks, were incubated with various concentrations of anti-DNP IgE (Sigma) for 20 hr in a CO_2_ incubator. The IgE sensitized cells were washed with 1% FBS-RPMI 1640 twice, and incubated with 100 ng/mL of DNP-HSA (Sigma) for 30 min at 37°C for activation, and then the reactions were stopped by placing them on ice. The cell suspension was then centrifuged for 800 x g for 5 min, and the supernatants were separated from the pellets. The pellets were lysed in lysis buffer containing 1% TritonX 100 (Sigma). The β-hexsosaminidase in supernatants and cell lysates were incubated with 32 mM citrate buffer (pH 4.5) and 2.8 mg/ml p-nitrophenyl N-acetyl-alpha-D-glucosaminide for 30 min at 37°C. The reactions were developed by adding 0.4 M glycine (pH 10.7), and the absorbance was measured at 405 and 570 nm using a multiplate reader. The percentage of β-hexosaminidase release was calculated as the percentage of the total β-hexosaminidase content. The histamine release and the content were measured by a pre-columned HPLC method as previously described [28].

### Western blotting

The following are proteins and the corresponding primary antibodies used for Western blotting: FcεRIβ (kind gift from Drs. Chisei Ra, Satoshi Nunomura, and Juan Rivera); FcεRIγ (anti-FcεRI γ subunit, EMD Millipore, Billerica, MA); tryptase (FL-275; Santa Cruz Biotechnology); and chymase (C-15; Santa Cruz Biotechnology); GAPDH (anti-GAPDH produced in rabbit, Sigma). Horseradish peroxidase-conjugated anti-mouse, anti-rabbit (GE Healthcare UK Ltd, England) and anti-goat immunoglobulin (Santa Cruz Biotechnology) were used as secondary antibodies. The 1×10^5^ cells (for tryptase and chymase detection) or 2×10^5^ cells (for β-chain and γ-chain detection) of 5–6-week cultured BMMC and FLMC were lysed in a cell lysis buffer containing NuPAGE LDS sample buffer (Invitrogen, Grand Island, NY), 50 µM dithiothreitol (Invitrogen), 10% β-mercaptoethanol (Kanto Chemical Co. Inc.), 200 µM benzamidine hydrochloride (Sigma), 200 µM sodium orthovanadate (Sigma), 1 mM sodium phosphate (Wako), and 10 mM sodium fluoride (Sigma), heated for 10 min at 70°C, and separated by electrophoresis on a 10% polyacrylamide gel containing 0.1% sodium dodecyl sulfate. Proteins were electrophoretically transferred onto a nitrocellulose or polyvinylidene difluoride membrane (Invitrogen), and Western blotting was performed according to the instructions for the Immobilon detection reagents (EMD Millipore). GAPDH was detected as a loading control for each sample. Briefly, the membranes were incubated in the stripping buffer (pH 6.7) containing 50 mM Tris (hydroxymethyl)-aminomethane (Sigma), 2% sodium dodecyl sulfate (Wako) and 0.75% β-mercaptoethanol (Sigma) for 30 min at 50°C, and washed twice with buffer (pH 6.7) containing 50 mM Tris (hydroxymethyl)-aminomethane (Sigma) and 2% sodium dodecyl sulfate (Wako). Next, re-probing was done using the anti-GAPDH antibody. Immunoreactive bands were visualized using an LAS-3000 imaging system (Fujifilm, Tokyo, Japan).

### Cytokine measurement

The culture periods and the sensitization of BMMC and FLMC is described in “Degranulation, histamine release and histamine content measurement”. The IgE-sensitized cells were washed twice with 1% FBS-RPMI 1640, and incubated with 100 ng/mL of DNP-HSA (Sigma) for 6 hr at 37°C for activation, and then the reactions were stopped by placing them on ice. The cell suspension was then centrifuged at 800 x g for 5 min, and the supernatants were separated from the pellets. The levels of cytokines in the culture supernatants were assayed by Flowcytomix (Bender MedSystems, Burlingame, CA) according to the manufacturer’s instructions.

### Isolation of RNA and generation of cDNA by reverse transcription (RT)

From 5–6-week cultured BMMC or FLMC, RNA was purified using the RNeasy mini kit (Qiagen, Valencia, CA) and was reverse-transcribed to cDNA according to the manufacturer’s instruction using an oligo (dT)_12–18_ primer, 10 mM deoxynucleotide triphosphate mix, 5 x first standard buffer, 0.1 M dithiothreitol, SuperScript^™^ II RNase H^-^ Reverse Transcriptase, and RNase H (all reagents from Invitrogen).

### RT-polymerase chain reaction (PCR)

PCR was carried out using AccuPrime SuperMix I (Invitrogen). Amplification was carried out for 40 cycles for mouse mast cell protease (mMCP) -1, mMCP-2, mMCP-4, mMCP-5, mMCP-6, mMCP-7, mMCP-8, mMCP-9 and mMCP-10. Each cycle included denaturation for 30 s at 94°C, annealing for 30 s (51°C for mMCP-1, mMCP-2, mMCP-4, mMCP-5, mMCP-7, mMCP-9 and mMCP-10; 55°C for mMCP-6 and mMCP-8) and extension for 1 min at 72°C. After the final cycle, the samples were incubated for 10 min at 72°C. PCR products were visualized by agarose gel electrophoresis followed by ethidium bromide staining. The primers used were as previously described [29] and were synthesized by Hokkaido System Science.

### Transcriptome analysis

Total RNA from BMMC and FLMC cultured for 6 weeks, isolated using the RNeasy mini kit (Qiagen) were qualified and quantified with the Agilent 2100 Bioanalyzer (Agilent Technologies, Santa Clara, CA) using the RNA 6000 kit (Agilent Technologies). Libraries of mRNA were prepared following the Illumina TruSeq RNA sample prep kit protocol. The DNA concentration of the cDNA library was measured using a 2100 Bioanalyzer with the DNA 1000 kit (Agilent). The library was sequenced by 100-bp single read using the Illumina Genome Analyzer IIx (Illumina, San Diego, CA).

Sequenced mRNA reads were mapped to the reference genome (UCSC mm9 gene) using standard mapping programs, Bowtie (0.12.7) and TopHat (Ver2.0.0). Next, differential expression of annotated genes identified by Bowtie or TopHat was tested using Cuffdiff, a program component of the Cufflinks package for testing differential gene expression.

### Statistical analysis

Statistical significance was determined using the two-tailed unpaired Student’s *t*-test for comparison between two groups, or the Student Newman Keuls test for comparison among three or more groups. Differences were considered significant at the P<0.05 level. Data are expressed as mean ± standard error of the mean (SEM).

## Results

### Surface expression of Fc epsilon RI and c-Kit on FLMC and BMMC

**Figure 1:**
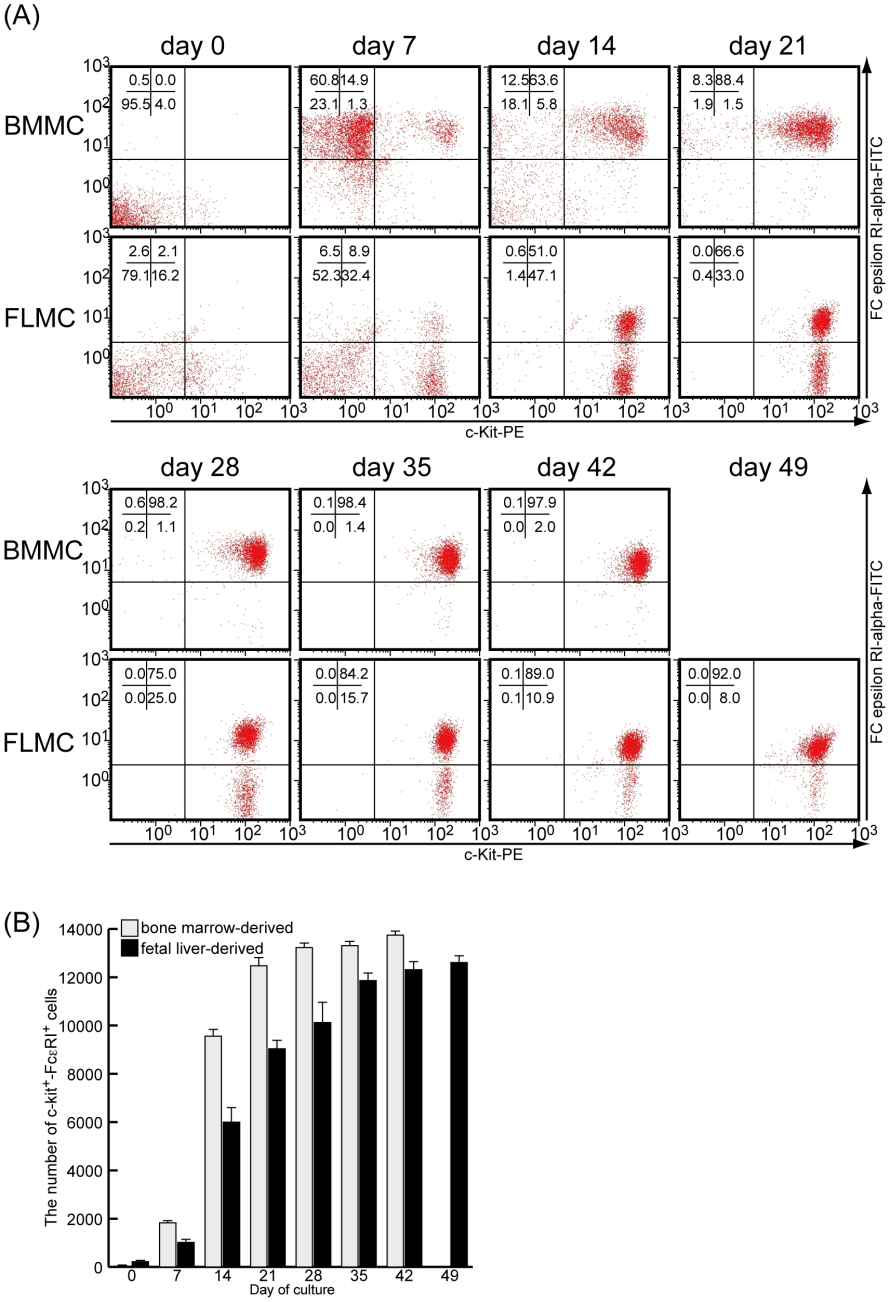
Surface expression of Fc epsilon RI and c-Kit on FLMC and BMMC. BMMC and FLMC were stained with FITC-conjugated anti-FcεRI Ab and PE-conjugated anti c-Kit Ab, and analyzed by flow cytometry. The live cells, with a rate of over 95% mast cells, were gated and 14,000 cells/sample were analyzed, and the expression of both c-Kit and FcεRI was observed. The surface expression of c-Kit occurred prior to the surface expression of FcεRI on FLMCs, although the surface expression of c-Kit on BMMC came later than FcεRI expression. At 6 weeks culture, the proportion of FcεRI-c-Kit double-positive cells was about 90% in FMLC (A). The results are representative of three independent experiments (A). The number of FcεRI-c-Kit double-positive cells increased sharply from day 14 in both BMMC and FLMC. However, the rate of increase was greater in BMMC than in FLMC. The bar graph expresses mean ± S.E of three independent experiments (B).

It is well known that mature mast cells express both FcεRI and c-Kit on the cell surface, and both molecules are pivotal for mast cell activation and differentiation. We first examined the time course of surface expression of the FcεRI alpha chain (FcεRIα) and c-Kit on BMMC and FLMC. The surface expression of c-Kit on BMMC was hardly observed until day 7, whereas the expression of FcεRIα on BMMC was clearly observed at day 7 with continuous expression until at least day 42. The ratio of FcεRIα/c-Kit double-positive cells to total BMMC reached 90% or more on day 28 (Figure 1). On FLMC, the surface expression of c-Kit preceded the appearance of FcεRIα; almost 95% of the total cells were c-Kit positive at day 14. Moreover, the overall proportion of FcεRIα/c-Kit double-positive cells among FLMC reached 90% or more on day 49 (Figure 1A). The number of FcεRI-c-Kit double-positive cells increased sharply from day 14 in both BMMC and FLMC. The rate of increase was greater in BMMC than FLMC, and the number of double-positive cells in BMMC was larger than in FLMC throughout the culture period (Figure 1B).

### Characteristics of lin^-^ cells from bone marrow and fetal liver

**Figure 2:**
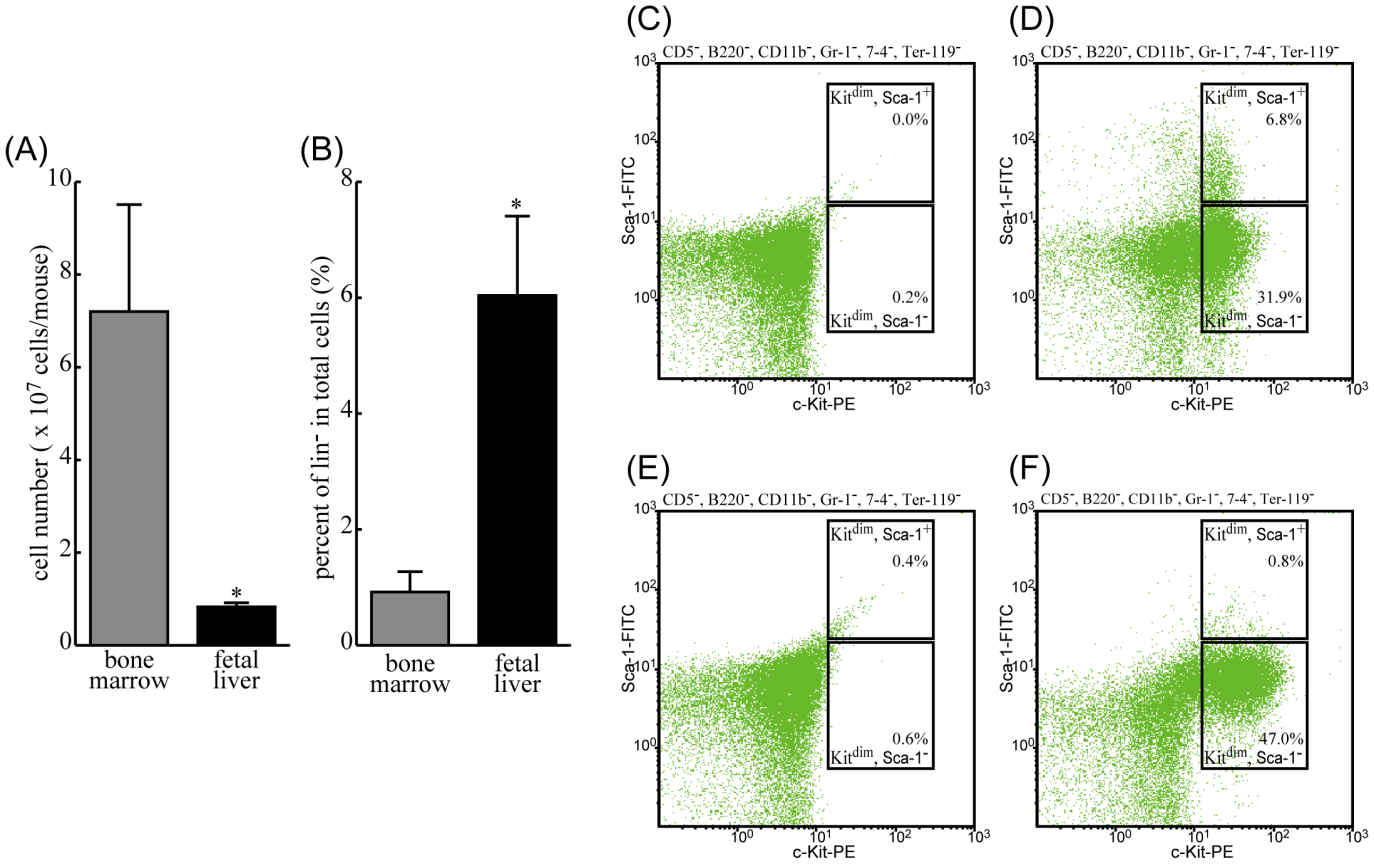
Comparison between characteristics of putative mast cell progenitors in BMMC and FLMC. The cells were freshly isolated from bone marrow or fetal liver, and suspended in culture medium as described in Materials and Methods. The numbers of cells were counted using a counting chamber (A). These cells were labeled with antibodies against lineage markers (CD5, B220, CD11b, Gr-1, 7–4, Ter-119), and fractionated with a magnetic cell sorting kit. The effluents were collected as lineage negative (lin^-^) cells, and the numbers of lin^-^ cells were counted using a counting chamber (B). The lin- cells were then stained with both PE-conjugated anti-c-Kit antibody and FITC-conjugated anti-Sca-1 antibody, or their respective isotype control IgG. 31.9% of c-Kit^dim^-Sca-1^-^ cells were observed in lin^-^ cells from bone marrow (D), and 47% of c-Kit^dim^-Sca-1^-^ cells were observed in lin^-^ cells from fetal liver (F). The isotype control-stained lin^-^ cells from bone marrow and from fetal liver are indicated in panels (C) and (E). The bar graphs express mean ± S.E of three independent experiments, and the histograms are representative of three independent experiments.

Although mast cell lineage characterization to date has been controversial, the putative precursor for mast cells occurs within a population of lin^-^ cells characterized as CD5^-^, B220^-^, CD11b^-^, Gr-1^-^, 7–4^-^ and Ter-119^-^. Moreover, it has been reported that mast cell progenitors might be present in the c-Kit-positive and Sca-1-positive/negative fraction of lin^-^ [30]–[32]. We investigated the ratio of lin^-^ cells and both c-Kit and Sca-1 expression on lin^-^-bone marrow cells and lin^-^-fetal liver cells. Figure 2A indicates the total number of cells prepared from one mouse, and Figure 2B indicates the percentile of lin^-^ cells among all of the cells prepared. The number of bone marrow cells was about 8 times higher than fetal liver cells. However, interestingly, the proportion of lin^-^ cells in fetal liver is about 7 times higher than in the bone marrow. Therefore, the total number of lin^-^ cells prepared from each tissue of any one mouse is almost the same. The ratio of c-Kit^dim^ and Sca-1^-^ cells in fetal liver was approximately 47% (Figure 2F), much higher than in bone marrow at roughly 32% (Figure 2D). In bone marrow, a relatively high rate of c-Kit^dim^ and Sca-1^+^ cells were observed, whereas these cells were hardly detected in fetal liver. Also, the fluorescence intensities derived from c-Kit-PE on c-Kit^dim^ cells were a little bit higher in fetal liver cells.

### Expression of mouse mast cell proteases (mMCP) in FLMC and BMMC

**Figure 3:**
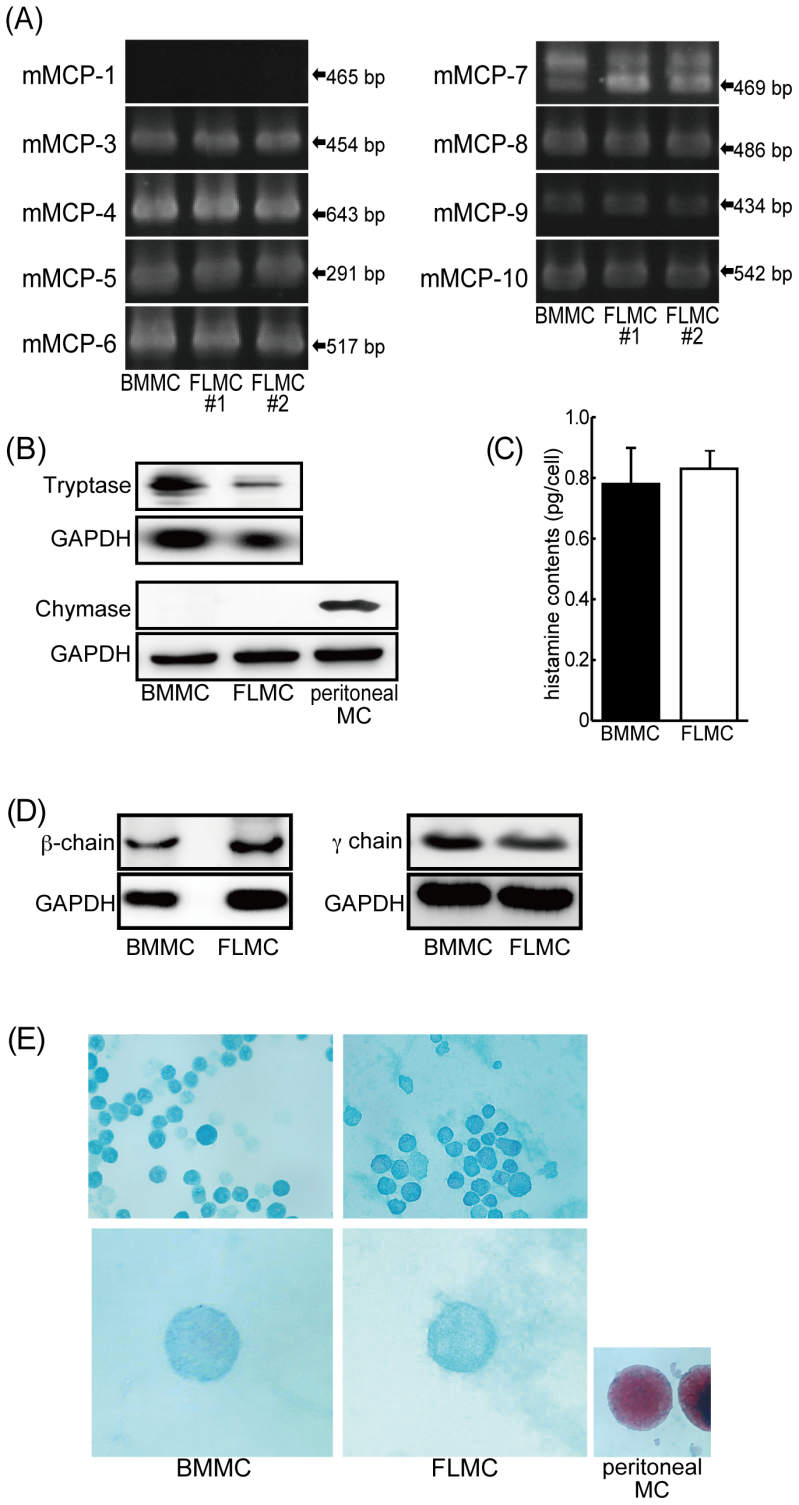
Comparison of properties of FLMC with BMMC. Mouse mast cell protease (mMCP) mRNA expression was assessed by RT-PCR (A). All but mMCP-1 mRNA expression was detected both on FLMC and BMMC. Chymase and tryptase expression was detected by Western blotting (B). Both mast cell types express tryptase although chymase was observed in neither FLMC nor BMMC. The results are representative of three independent experiments. Histamine content was measured by HPLC (C). Histamine content was almost the same in both FLMC and BMMC, and the amount of histamine contained in both FLMC and BMMC was about 0.8 pg/cell. Data represent the mean ± SEM of three to five experiments. The components of FcεRI in FLMC were compared with BMMC (D). As with BMMC, FcεRI on FLMC had both a beta chain and a gamma chain. The results are representative of three independent experiments. FLMC and BMMC were stained by both safranin O and alcian blue (E). The granules in mast cells were densely stained by alcian blue; these granules were lightly but not densely stained by safranin O compared with mast cells isolated from peritoneal fluid of C57BL/6 mice. The representative photos are 5-week cultured. The results are representative of three independent experiments.

A wide variety of mMCP are known to be expressed in BMMC [29]. We investigated the mRNA expression of mMCP in FLMC and BMMC using conventional PCR. In BMMC, although mMCP-1 mRNA was not detected, the expression of mRNAs for mMCP-3, mMCP-4, mMCP-5, mMCP-6, mMCP-7, mMCP-8, mMCP-9 and mMCP-10 were observed. These results for BMMC are the same as those previously reported [29]. In FLMC, mRNA expression was observed for mMCP-3, mMCP-4, mMCP-5, mMCP-6, mMCP-7, mMCP-8, mMCP-9 and mMCP-10; these results are identical to those for BMMC (Figure 3A). We next examined chymase and tryptase expression in BMMC and FLMC by Western blotting. Although chymase was not expressed, tryptase was clearly detected in both mast cell types (Figure 3B). Chymase was also detected in peritoneal mast cells (positive control, Figure 3B).

### Comparison of the FcεRI subunit in BMMC and FLMC

It is well known that the high affinity IgE receptor, abundantly expressed on mast cells, comprises a complex of four components: an alpha chain, a beta chain and two gamma chains. We examined the expression levels of these subunits in BMMC and FLMC. The beta chain and the gamma chain were detected in both BMMC and FLMC by Western blotting (Figure 3D). Surface expression of the alpha chain was observed as previously described (Figure 1).

### The staining properties of FLMC and BMMC

The granules within different types of mast cells such as serosal-type or mucosal-type mast cells are known to have distinct properties with regard to staining for safranin O or alcian blue. BMMC and FLMC were stained with both safranin O and alcian blue and the granules were equally stained in both cases (Figure 3E). Mouse peritoneal mast cells, a positive control, were clearly stained by safranin O alone (Figure 3E).

### Transmission electron microscopy

**Figure 4:**
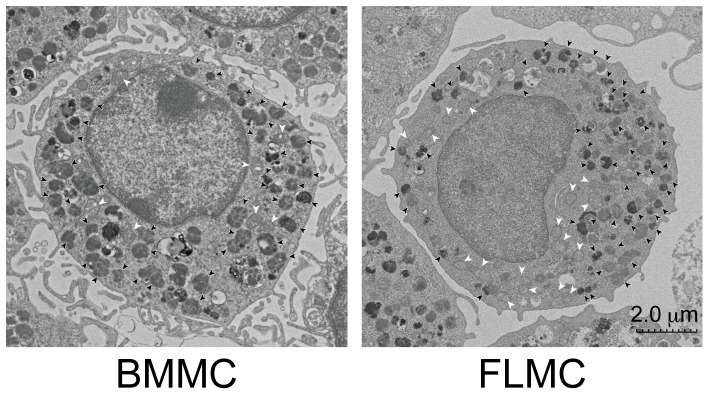
TEM of FLMC and BMMC. FLMC and BMMC were observed by transmission electron microscopy. The black arrow indicates mast cell granules and the white arrow indicates mitochondria. Both mast cells had a lot of microvilli on surface of the cells. The nucleus in both cells resembled the shape of that in mononuclear cells, but not in polymorphonuclear leucocytes. The granules in FLMC and BMMC were both slightly smaller and the electron density was not so high; these characteristics appeared to be similar to that of mucosal-type mast cells. The electron density of the granules in FLMC was lower than in BMMC. The photographs are representative of fifty or more photographs of BMMC or FLMC.

BMMC and FLMC were observed by transmission electron microscopy to examine the detailed internal structure of both mast cell types. By TEM, a number of microvilli were observed on the surface of both BMMC and FLMC. Additionally, numerous small granules were observed in the cytoplasm of both BMMC and FLMC, with the number of granules in FLMC (55.6±4.9/cell) statistically higher than in BMMC (41.5±3.4/cell). While the granules in FLMC were similar in size to those in BMMC (data not shown), the number of mitochondria in FLMC appeared to be slightly higher than in BMMC (Figure 4).

### Histamine content, degranulation and histamine release in FLMCs and BMMCs in response to IgE cross-linking

**Figure 5:**
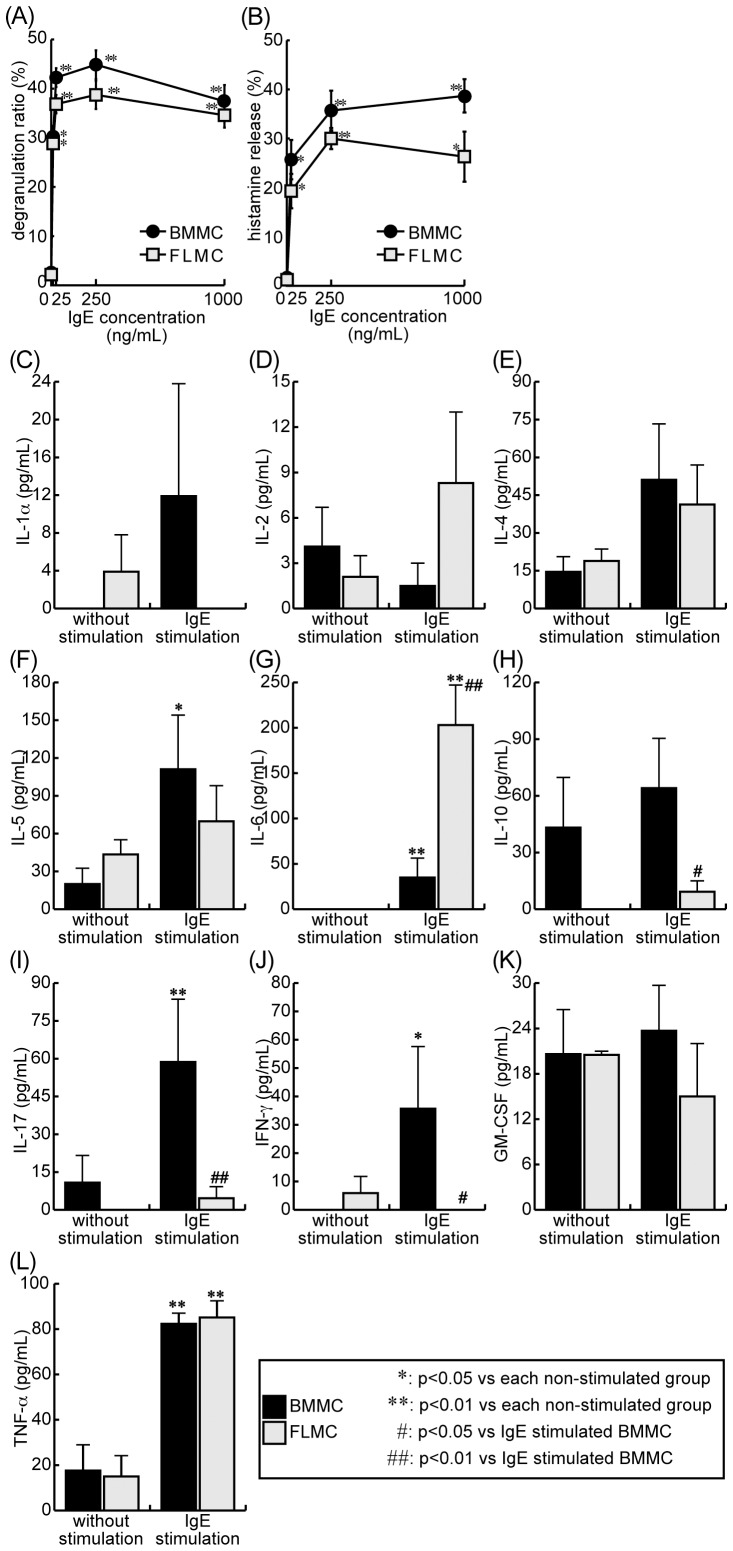
Degranulation ratio, histamine release and cytokine generation from FLMC and BMMC. Both FLMC and BMMC were stimulated by DNP-HSA following sensitization of anti-DNP IgE as described in Materials and Methods. Degranulation caused by FcεRI cross-linking was measured by β-hexosaminidase assay (A). Histamine release by FcεRI stimulation was measured by HPLC (B). Degranulation ratio and histamine release were almost the same in both FLMC and BMMC. Cytokine generation after the stimulation was measured by flow cytomix (C–L). Although IL-6 production (G) in FLMC was significantly larger than that in BMMC, little IL-17 (I) and IFN-γ (J) was generated in FLMC. Production of the other cytokines showed no significant difference between FLMC and BMMC. *: *p*<0.05, **: *p*<0.01 vs. no added IgE of each cells. #: *p*<0.05, ##: *p*<0.01 vs. IgE stimulated BMMC. Data represent the mean ± SEM of three to six experiments.

Degranulation following IgE cross-linking was assessed by the β-hexosaminidase assay. The maximum degranulation ratio reached about 42% in BMMC and about 38% in FLMC (Figure 5A). The maximum degranulation ratios in both cell types were observed when the cells were treated with 250 ng/mL of IgE. At all of the IgE concentrations used, the degranulation ratio in BMMC tended to be higher than in FLMC, but this was not statistically significant (Figure 5A). The treatment of BMMC with 1,000 ng/mL of IgE caused the maximum rate of histamine release, about 40%. On the other hand, stimulation with 250 ng/mL IgE induced a maximum histamine release in FLMC of about 30% (Figure 5B). As with the degranulation ratio, the ratio of histamine release in BMMC tended to be higher than in FLMC, but this also was not statistically significant at all of the IgE concentrations used (Figure 5B).

The histamine content in FLMC was about 0.8 pg/cell, which was almost the same as that in BMMC (Figure 3C).

### Cytokine generation following FcεRI cross-linking

Mast cells are known to generate various cytokines by FcεRI stimulation. We observed cytokine production following FcεRI cross-linking on these cells, and compared FLMC and BMMC. As shown in Figure 5, FcεRI stimulation caused generation of IL-1α, IL-4, IL-5, IL-6, IL-17, IFN-γ and TNF-α in BMMC. In contrast, IL-1α, IL-17 and IFN-γ generation was not observed in FLMC. Large quantities of IL-6 were produced by FLMC (Figure 5C–L).

### Transcriptome comparison of BMMC and FLMC

**Figure 6:**
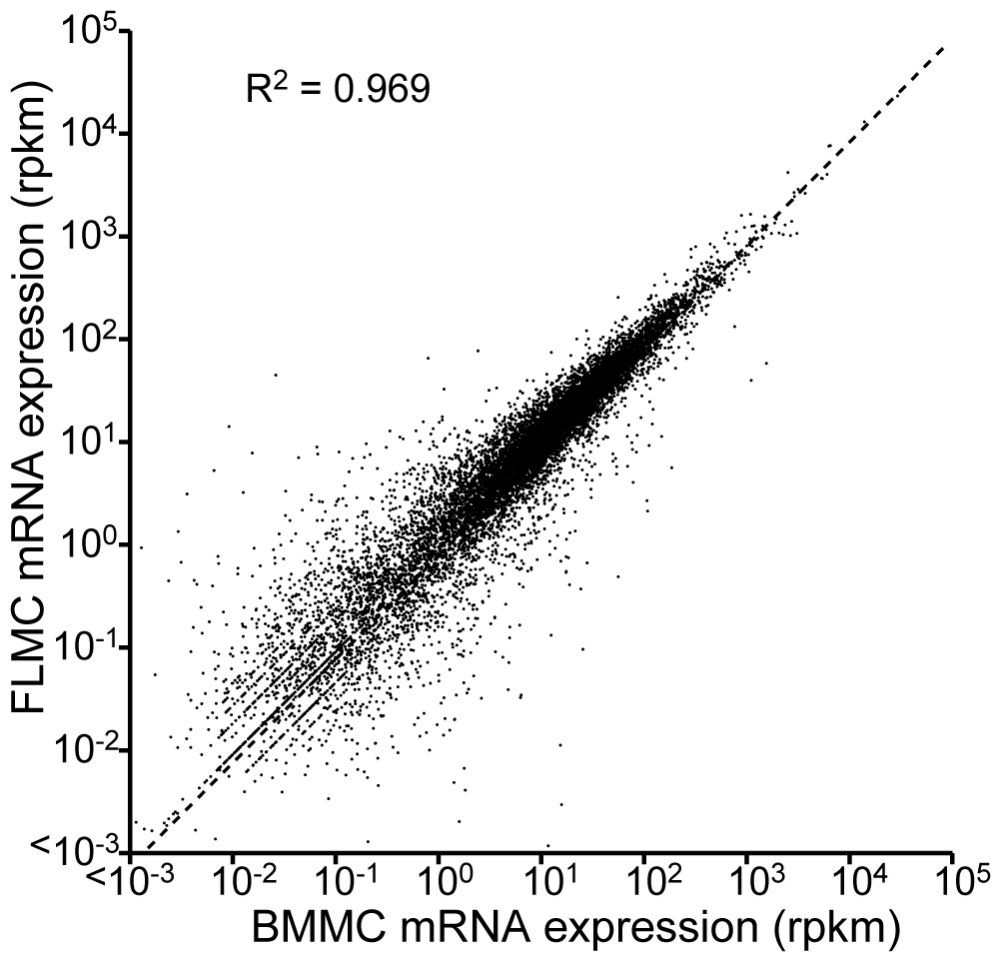
Comparison of genes expressed by FLMC and BMMC. The expression levels of 28,800 pairs of identical genes were compared. The Y axis represents transcript level on FLMC, and the X axis indicates those on BMMC. The square value of Pearson product-moment correlation coefficient was 0.969, indicating that these expression levels showed a strong correlation.

**Figure 7:**
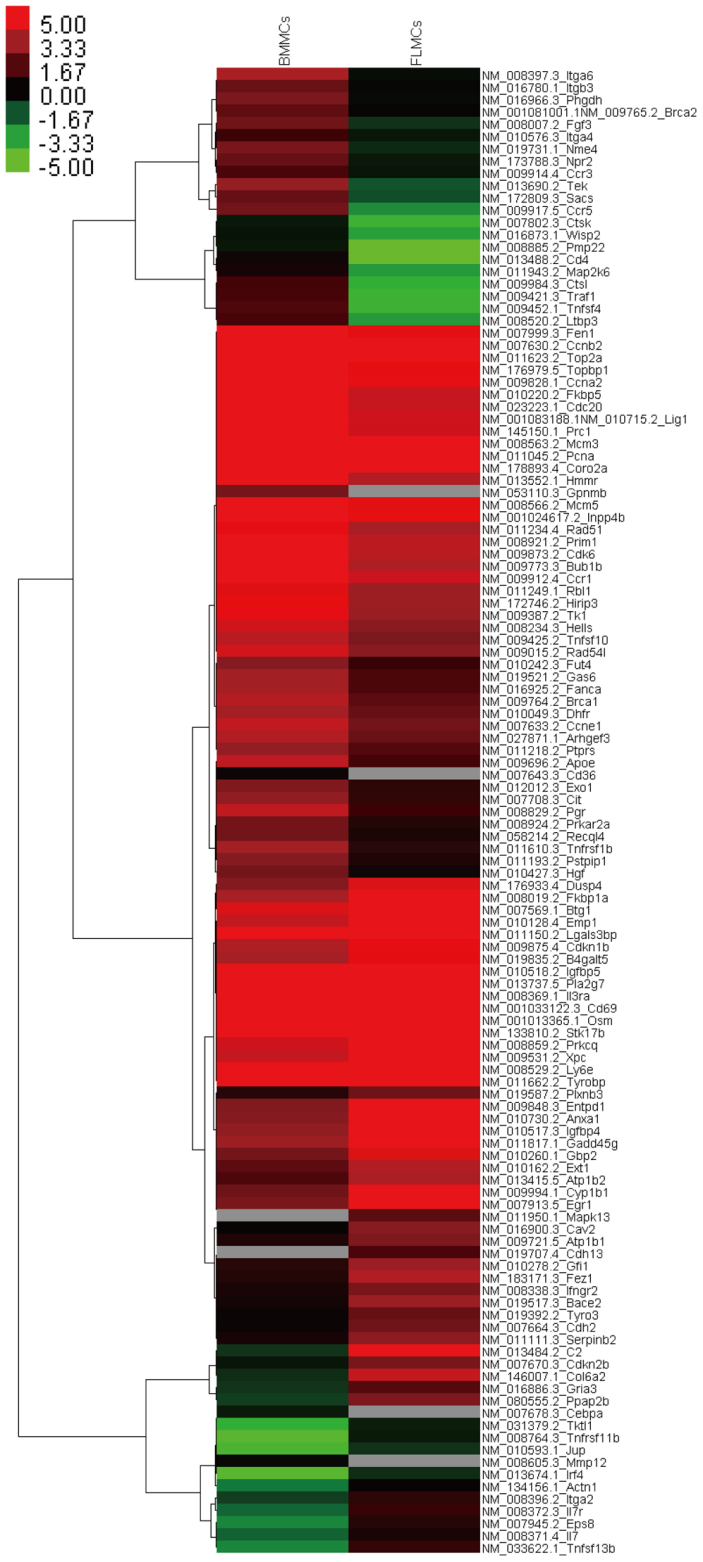
mRNA expression comparison between BMMC and FLMC. mRNA expression showing statistically significant differences between BMMC and FLMC are displayed as a heat map colored according to the expression level.

We compared gene expression profiles between BMMC and FLMC. In this transcriptome analysis, we could detect 22,800 transcripts in both mast cell types. The expression of about 22,500 transcripts showed no significant difference, which was 98.6% of the analyzable mRNAs in this experiment. The coefficient of correlation between both transcripts levels in BMMC and FLMC was about 0.96 (Figure 6). The mRNAs which were reported to encode functional proteins and which were observed to display significantly different expression patterns between BMMC and FLMC are summarized as a heat map (Figure 7). Additionally, the transcripts for chemokines, cytokines, their receptors and other mast cell-related proteins expressed in BMMC and FLMC are listed in Table 1. Among these transcripts, significantly different expression levels were observed for C-C chemokine 2 (0.39-fold), C-C chemokine receptor 1 (0.28-fold), C-C chemokine receptor 3 (0.26-fold), C-C chemokine receptor 5 (0.03-fold), C-C chemokine receptor 8 (10-fold), interleukin 7 (5.9-fold), interferon gamma receptor 2 (3.8-fold), interleukin 3 receptor, alpha chain (2.2-fold), interleukin 7 receptor (9.2-fold), Fc gamma III (2.6-fold), leukotriene B4 receptor 1 (3.9-fold) and complement C3a receptor 1 (2.2-fold).

**Table 1:** Transcripts for chemokines, cytokines, and their receptors expressed byBMMC and FLMC.

Function	Name of transcript	Genename	Accession No	relative expression		q value	significance
				BMMCs	FLMCs		
Chemokines							
	Chemokine (C-C motif) ligand 1	Ccl1	NM_011329.3	2.8	3.34	9.55E-01	no
	Chemokine (C-C motif) ligand 2	Ccl2	NM_011333.3	164.86	64.82	9.73E-03	yes
	Chemokine (C-C motif) ligand 3	Ccl3	NM_011337.2	8.5	4.58	5.99E-01	no
	Chemokine (C-C motif) ligand 4	Ccl4	NM_013652.2	14.46	25.73	5.41E-01	no
	Chemokine (C-C motif) ligand 7	Ccl7	NM_013654.2	0.38	0.69	7.78E-01	no
	Chemokine (C-C motif) ligand 9	Ccl9	NM_011338.2	17.8	16.59	9.59E-01	no
	Chemokine (C-C motif) ligand 17	Ccl17	NM_011332.2	5.56	3.11	7.01E-01	no
	Chemokine (C-C motif) ligand 24	Ccl24	NM_019577.4	0.41	0	1.06E-01	no
	Chemokine (C-X-C motif) ligand 16	Cxcl16	NM_023158.6	0.89	1.78	5.51E-01	no
	Chemokine (C-X3-C motif) ligand 1	Cx3cl1	NM_009142.3	0.75	0.18	1.05E-01	no
Chemokine receptors							
	Chemokine (C-C motif) receptor 1	Ccr1	NM_009912.4	69.48	19.41	4.14E-05	yes
	Chemokine (C-C motif) receptor 2	Ccr2	NM_009915.1	2.56	7.12	5.70E-02	no
	Chemokine (C-C motif) receptor 3	Ccr3	NM_009914.4	2.89	0.76	4.55E-02	yes
	Chemokine (C-C motif) receptor 5	Ccr5	NM_009917.5	5.13	0.14	2.29E-09	yes
	Chemokine (C-C motif) receptor 8	Ccr8	NM_007720.2	0.24	2.37	4.76E-03	yes
	Chemokine (C-X3-C) receptor 1	Cx3cr1	NM_009987.3	75.02	68.11	9.33E-01	no
Cytokines							
	Colony stimulating factor 1	Csf1	NM_007778.3	3.75	6.22	4.96E-01	no
	Interleukin 1b	Il1b	NM_008361.3	ND	ND	—	---
	Interleukin 4	Il4	NM_021283.2	ND	ND	---	---
	Interleukin 6	Il6	NM_031168.1	48.99	54.66	9.33E-01	no
	Interleukin 7	Il7	NM_008371.4	0.25	1.45	2.17E-02	yes
	Interleukin 13	Il13	NM_008355.2	6.99	19.8	6.90E-02	no
	Interleukin 15	Il15	NM_008357.1	9.15	16.03	4.97E-01	no
	Interleukin 16	Il16	NM_010551.3	15.47	20.08	7.11E-01	no
Cytokine receptors							
	Interferon (alpha and beta) receptor 1	Ifnar1	NM_010508.2	9.23	8.69	9.63E-01	no
	Interferon (alpha and beta) receptor 2	Ifnar2	NM_010509.1	6.78	7.49	9.56E-01	no
	Interferon gamma receptor 1	Ifngr1	NM_010511.2	14.15	13.98	9.94E-01	no
	Interferon gamma receptor 2	Ifngr2	NM_008338.3	1.46	5.6	3.82E-02	yes
	Colony stimulating factor 2 receptor, beta 2, low-affinity (granulocyte-macrophage)	Csf2rb2	NM_007781.2	2873.06	3154.06	9.75E-01	no
	Interleukin 1 receptor, type I	Il1r1	NM_008362.2	0	0	1.00E+00	no
	Interleukin 2 receptor, alpha chain	Il2ra	NM_008367.2	2.79	6.56	1.08E-01	no
	Interleukin 2 receptor, gamma chain	Il2rg	NM_013563.3	141.56	204.96	4.68E-01	no
	Interleukin 3 receptor, alpha chain	Il3ra	NM_008369.1	35.37	77.86	3.64E-02	yes
	Interleukin 4 receptor, alpha	Il4ra	NM_001008700.3	335.93	256.25	7.62E-01	no
	Interleukin 5 receptor, alpha	Il5ra	NM_008370.2	1.48	1.35	9.70E-01	no
	Interleukin 7 receptor	Il7r	NM_008372.3	0.24	2.17	7.18E-04	yes
	Interleukin 9 receptor	Il9r	NM_008374.1	1.66	1.92	9.56E-01	no
	Interleukin 10 receptor, alpha	Il10ra	NM_008348.2	69.01	77.96	9.10E-01	no
	Interleukin 10 receptor, beta	Il10rb	NM_008349.4	13.88	20.77	5.78E-01	no
	Interleukin 11 receptor, alpha chain 1	Il11ra1	NM_010549.2	3.56	1.7	4.65E-01	no
	Interleukin 12 receptor, beta 1	Il12rb1	NM_008353.2	6.34	8.63	7.62E-01	no
	Interleukin 12 receptor, beta 2	Il12rb2	NM_008354.3	19.93	24.72	8.08E-01	no
	Interleukin 15 receptor, alpha chain	Il15ra	NM_008358.1 NM_133836.1	2.27	4.4	5.61E-01	no
	Interleukin 17 receptor A	Il17ra	NM_008359.1	39.45	36.88	9.57E-01	no
	Interleukin 18 receptor 1	Il18r1	NM_008365.1	1.3	0.46	1.28E-01	no
	Interleukin 20 receptor beta	Il20rb	NM_001033543.3	0.95	0.57	7.52E-01	no
	Interleukin 21 receptor	Il21r	NM_021887.1	3.5	1.54	3.34E-01	no
	Interleukin 27 receptor, alpha	Il27ra	NM_016671.2	22.7	10.98	9.88E-02	no
Others							
	Kit oncogene	Kit	NM_021099.2	587.33	523.55	8.92E-01	no
	Fc receptor, IgE, high affinity I, alpha polypeptide	Fcer1a	NM_010184.1	7566.7	6278.82	9.25E-01	no
	Fc receptor, IgE, high affinity I, gamma polypeptide	Fcer1g	NM_010185.4	1115.22	1227.03	9.40E-01	no
	Fc receptor, IgG, high affinity I	Fcgr1	NM_010186.5	0.33	1.4	6.78E-02	no
	Fc receptor, IgG, low affinity IIb	Fcgr2b	NM_001077189.1 NM_010187.2	15.12	11.34	8.23E-01	no
	Fc receptor, IgG, low affinity III	Fcgr3	NM_010188.4	83.32	217.62	2.43E-03	yes
	Prostaglandin E receptor 1 (subtype EP1)	Ptger1	NM_013641.2	32.25	31.39	9.84E-01	no
	Prostaglandin E receptor 3 (subtype EP3)	Ptger3	NM_011196.2	50.74	57.46	9.11E-01	no
	Prostaglandin E receptor 4 (subtype EP4)	Ptger4	NM_008965.1	11.69	12.43	9.69E-01	no
	Prostaglandin I receptor (IP)	Ptgir	NM_008967.2	12.04	5.95	1.63E-01	no
	Leukotriene B4 receptor 1	Ltb4r1	NM_008519.1	9.23	35.9	1.74E-03	yes
	Leukotriene B4 receptor 2	Ltb4r2	NM_020490.1	4.09	11.69	9.70E-02	no
	Complement component 3a receptor 1	C3ar1	NM_009779.2	21.72	48.69	1.69E-02	yes
	Complement component 5a receptor 1	C5ar1	NM_007577.3	0.26	1.09	9.63E-02	no

## Discussion

Intra-utero lethality caused by gene deletion often interferes with the functional analysis of gene products [15]. RNA interference including small interfering RNA [33] and short hairpin RNA [34], and the construction of genetically modified cell lines [35] are among some of the promising approaches to overcome this difficulty. Although small hairpin RNA technology using retrovirus vectors is a useful procedure for silencing of target transcripts, small interfering RNAs are, in many cases, barely effective in mast cells. Construction of genetically modified cell lines is a hopeful approach. Yamazoe *et al* demonstrated that the chicken B lymphocyte line DT40, which is genetically modulated by avian leucosis virus, is a beneficial tool for understanding cellular functions [36]. Also, Adach *et al* indicated that a human pre-B cell line Nalm-6 is useful for gene targeting by homologous recombination [37], [38]. However, the application of this technique is quite restrictive, and only a chicken cell line and human pre-B cell line are applicable at present.

We generated mast cells from liver cells of embryos at embryonic age 12.5 days, and these cells were degranulated by IgE cross-linking, as for BMMC. Nakajima *et al* compared the expression of chemokines, cytokines, and their receptors between human mast cells and BMMC [39]. They reported that the relative expression of CCL2, IL-6, IL-16, IL-3 receptor, IL-4 receptor and c-Kit in the resting BMMC was greater than for the other chemokines, cytokines and their receptors. Additionally, the functions of mast cells were reported to be subject to regulation by fractalkine [40], GM-CSF [41], IL-2, IL-10 [42], [43], IL-12 [44], prostaglandin E2 [45], [46] and C3a [8]. Our results indicate that the transcripts for CCL2, IL-6, IL-16, IL-3 receptor, IL-4 receptor and c-Kit on BMMC are also relatively higher than the others, and this tendency was also observed in FLMC. Additionally, our results also show that the mRNA expression of CCL7, CCL9, CCR1, Cx3CR1 (fractalkine receptor), GM-CSF receptor, IL-2 receptor, IL-10 receptor, IL-12 receptor, IL-17 receptor A, IL-27 receptor, FcεRI, FcγRIII, EP1, EP3 and C3a receptor are high in both BMMC and FLMC. Therefore, as for BMMC, FLMC seem to have the potential to be useful for the investigation of mast cells in response to microenvironmental insult such as cytokine and prostaglandins. In this study, we compared the potential for cytokine generation in these cell types. However, we could not identify a pattern for the differences in cytokine generation between BMMC and FLMC. For example, IL-4, IL-5, IL-6, IL-10 and TNF-α are well known as the common cytokines generated by mast cells, and mast cells are known to generate IL-4 in response to “weak stimulation” by IgE-bound FcεRI, while these cells also are known to generate IL-6 and IL-10 under “strong stimulation”. However, there is no specific pattern for generating these cytokines; IL-5 generation in BMMC is higher than in FLMC but IL-6 generation in BMMC is lower than in FLMC, while IL-4 and TNF-α generation is almost the same in both cell types.

The classification of mouse mast cells has been based on the biochemical and functional differences between connective tissue mast cells (CTMC) and mucosal mast cells (MMC) [47]. The FLMC seem best classified as MMC, as for BMMC. The heterogeneity of the differentiation process associated with a different source of progenitor cells has not been fully discussed, although the mechanisms of differentiation and/or diversity of the phenotype arising from the progenitors have been well discussed for mast cells [48], [49]. In the present study, we demonstrated that although FcεRI surface expression was observed ahead of c-Kit expression in bone marrow-derived cells, the order of the expression onset was reversed in fetal liver-derived cells. On the other hand, FLMC revealed almost the same phenotype as BMMC following 4 weeks of culture: histamine content, degranulation ratio, expression profile of mast cell proteases, alcian blue/safranin O staining properties and constitutive FcεRI subunit expression were the same in BMMC and FLMC. Arinobu *et al* demonstrated that common lymphoid progenitors characterized as lin^-^ Sca-1^low^ c-Kit^low^ IL-7α^+^, and common myeloid progenitors identified as lin^-^ Sca-1^-^ c-Kit^low^ IL-7α^+^ are both present in bone marrow cells, and showed that common myeloid progenitors but not common lymphoid progenitors are capable of generating mast cells [49]. In the present study, both approximately 32% of lin^-^ c-Kit^dim^ Sca-1^-^ in bone marrow and 47% of these cells in fetal liver were observed; the candidate mast cell progenitor may exist in both cell populations. However, the fluorescence intensity caused by the PE-conjugated anti-c-Kit antibody on c-Kit^dim^ Sca-1^-^ cells was a little bit higher in fetal liver cells compared than in bone marrow, although cell size estimated by forward scatter is almost the same. These findings indicate lin^-^ c-Kit^dim^ Sca-1^-^ cells in bone marrow cells might be different from those in fetal liver in terms of differentiation stage, implying that phenotypically similar mast cells may not undergo the same pathways of differentiation.

Stem cell factor, well known as c-Kit ligand, has been reported to affect the development, survival and activation of mast cells. In FLMC, the surface c-Kit expression was observed from 7 days of culture, unlike in BMMC. Therefore, using both fetal liver- and bone marrow-derived cells, we observed whether SCF influences the early stage of the growth of mast cells. When the cells were cultured in complete RPMI supplemented with 5 ng/ml of IL-3 and 50 ng/ml of SCF from day 7, cell viability, c-Kit and FcεRI expression on FLMC at day 42 were the same as for BMMC. Additionally, electron microscopic findings indicated that the density of mast cell granules was slightly higher in FLMC cultured with SCF compared to without SCF (Figure S1). These results suggest that the development of murine mast cells is not markedly affected by the addition of SCF, at least in the early stage of expansion. Further functional studies will be needed to elucidate the effects of SCF at different stages of mast cell differentiation.

Recent studies have indicated that binding of FcεRI to monomeric IgE plays an important role in not only the function but also the differentiation of mast cells [48], [50]. Lam *et al* have demonstrated that IgE stimulates mast cell adhesion to fibronectin [51]. Also, Tanaka *et al* have clarified that IgE increases histamine synthesis and IL-6 generation through Ca^++^ influx without antigen stimulation [52]. Moreover, Kashiwakura *et al* have shown that mRNA expression patterns of mouse mast cell proteases and mast cell-related transcription factors are altered in mast cells sensitized with highly cytokinergic IgE compared to those cultured without IgE [53]. Therefore, the timing of surface FcεRI expression during the development period may be crucial for the determination of the functional fate of mast cells. The comparison between BMMC and FLMC may be beneficial to the investigation of mast cell fate arising from the timing of FcεRI expression.

Taken together, FLMC are a powerful tool for the elucidation of protein function in mast cells, even if gene manipulation triggers embryonic lethality, and they are potentially useful for revealing the processes underlying mast cell differentiation and proliferation.

## Supporting Information

Figure S1 **The expression of FcεRI and c-Kit and TEM of FLMC cultured with SCF.** During the first 7 days, fetal liver cells were cultured in RPMI1640 medium supplemented with 1 mM pyruvate, penicillin-streptomycin, non-essential amino acid, 10% fetal calf serum, 100 µg/ml 2-mercaptoethanol and 10% CM. From 8 days on, the cells were continuously cultured in RPMI 1640 medium supplemented with 1 mM pyruvate, penicillin-streptomycin, non-essential amino acids, 10% FBS, 100 µg/ml 2-mercaptoethanol, 5 ng/ml of interleukin-3 and 50 ng/ml of SCF. The histograms indicate FLMC at 42 days culture. Approximately 92% of the cell was c-Kit^+^ FcεRI^+^ cells (A). The photograph of transmission electron microscopy of FLMC at 35days culture (B). There is a lot of microbili on the cell surface, and many granules in cytosol are observed, as with FLMC cultured without SCF. Whereas, compared with FLMC cultured without SCF (see Figure 4), electron density of the granules in FLMC cultured with SCF is bit dense.
(TIF)
