# Supplementary figures and images for: Generation of Mast Cells from Mouse Fetus: Analysis of Differentiation and Functionality, and Transcriptome Profiling Using Next Generation Sequencer
Source: PLoS One. 2013 Apr 3;8(4):e60837. doi: 10.1371/journal.pone.0060837 (PMC3616098; doi:10.1371/journal.pone.0060837)

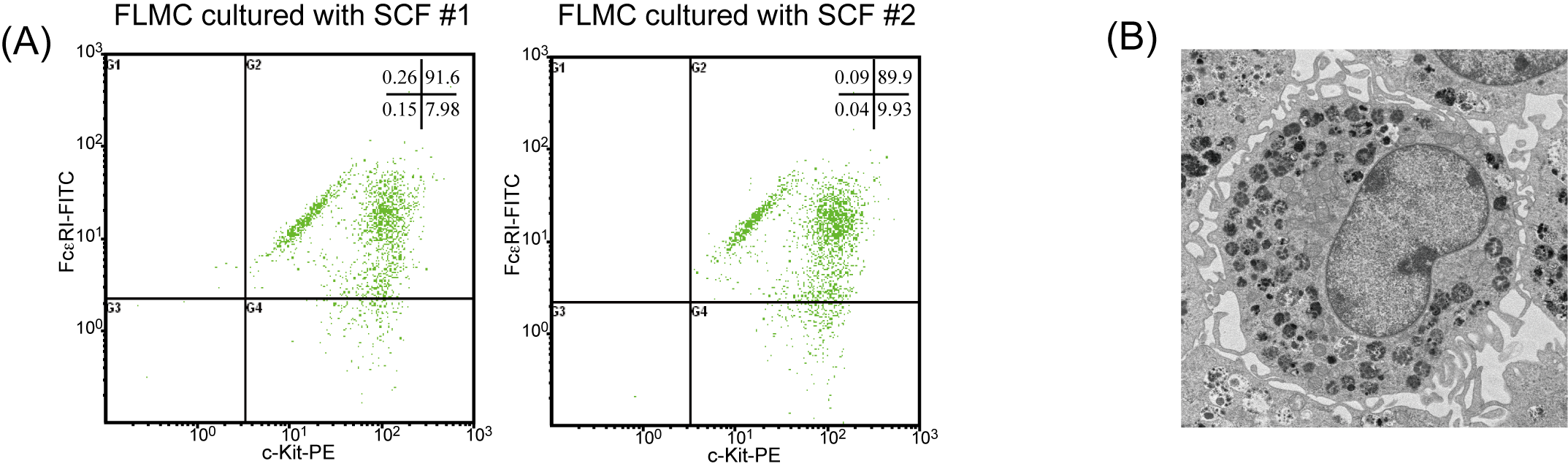

Supplement: Figure S1 — The expression of FcεRI and c-Kit and TEM of FLMC cultured with SCF. During the first 7 days, fetal liver cells were cultured in RPMI1640 medium supplemented with 1 mM pyruvate, penicillin-streptomycin, non-essential amino acid, 10% fetal calf serum, 100 µg/ml 2-mercaptoethanol and 10% CM. From 8 days on, the cells were continuously cultured in RPMI 1640 medium supplemented with 1 mM pyruvate, penicillin-streptomycin, non-essential amino acids, 10% FBS, 100 µg/ml 2-mercaptoethanol, 5 ng/ml of interleukin-3 and 50 ng/ml of SCF. The histograms indicate FLMC at 42 days culture. Approximately 92% of the cell was c-Kit+ FcεRI+ cells (A). The photograph of transmission electron microscopy of FLMC at 35days culture (B). There is a lot of microbili on the cell surface, and many granules in cytosol are observed, as with FLMC cultured without SCF. Whereas, compared with FLMC cultured without SCF (see Figure 4), electron density of the granules in FLMC cultured with SCF is bit dense. (TIF) [file pone.0060837.s001.tif]
